# Nucleocytoplasmic p27^Kip1^ Export Is Required for ERK1/2-Mediated Reactive Astroglial Proliferation Following Status Epilepticus

**DOI:** 10.3389/fncel.2018.00152

**Published:** 2018-06-07

**Authors:** Ji-Eun Kim, Tae-Cheon Kang

**Affiliations:** Department of Anatomy and Neurobiology, Institute of Epilepsy Research, College of Medicine, Hallym University, Chuncheon, South Korea

**Keywords:** CRM1, epilepsy, Ki-67, LMB, seizure, U0126, roscovitine

## Abstract

Reactive astrogliosis is a prominent and ubiquitous reaction of astrocytes to many types of brain injury. Up-regulation of glial fibrillary acidic protein (GFAP) expression and astroglial proliferation are hallmarks of reactive astrogliosis. However, the mechanisms that regulate reactive astrogliosis remain elusive. In the present study, status epilepticus (SE, a prolonged seizure activity) led to reactive astrogliosis showing the increases in GFAP expression and the number of proliferating astrocytes with prolonged extracellular signal receptor-activated kinases 1/2 (ERK1/2) activation and reduced nuclear p27^Kip1^ level. U0126, an ERK1/2 inhibitor, showed opposite effects. Leptomycin B (LMB), an inhibitor of chromosomal maintenance 1 (CRM1), attenuated nucleocytoplasmic p27^Kip1^ export and astroglial proliferation, although it up-regulated ERK1/2 phosphorylation and GFAP expression. Roscovitine ameliorated the reduced nuclear p27^Kip1^ level and astroglial proliferation without changing GFAP expression and ERK1/2 phosphorylation. U0126 aggravated SE-induced astroglial apoptosis in the molecular layer of the dentate gyrus that was unaffected by LMB and roscovitine. In addition, U0126 exacerbated SE-induced neuronal death, while LMB mitigated it. Roscovitine did not affect SE-induced neuronal death. The present data elucidate for the first time the roles of nucleocytoplasmic p27^Kip1^ transport in ERK1/2-mediated reactive astrogliosis independent of SE-induced neuronal death and astroglial apoptosis. Therefore, our findings suggest that nucleocytoplasmic p27^Kip1^ export may be required for ERK1/2-mediated astroglial proliferation during reactive astrogliosis, and that nuclear p27^Kip1^ entrapment may be a potential therapeutic strategy for anti-proliferation in reactive astrocytes.

## Introduction

Astrocytes are the most numerous non-neuronal cell types in the brain, which participate in the maintenance of extracellular glutamate level (Anderson and Swanson, 2000; Mazzanti et al., 2001), ionic/pH environment (Amiry-Moghaddam and Ottersen, 2003; Simard and Nedergaard, 2004), metabolic substrates (Kasischke et al., 2004), and brain-blood barrier integrity (Takano et al., 2006). Recent studies have revealed that astroglial subpopulations show a differential vulnerability in response to status epilepticus (SE, a prolonged seizure activity). Astrocytes are acutely degenerated in the molecular layer of the dentate gyrus, but not in the CA1 region (Kang et al., 2006; Kim et al., 2010, 2011, 2014a, 2017). These regional specific astroglial death patterns reflect functional heterogeneity of astrocytes. Regardless of considering SE-induced astroglial damage, surviving astrocytes in the affected region become hypertrophic and proliferate, a process termed reactive astrogliosis (Ridet et al., 1997). Reactive astrocytes play complex and potentially biphasic roles after brain injury. Astroglial scar formation inhibits dendritic and axonal remodeling in neuronal circuits (Horner and Gage, 2000; Rossi et al., 2007). Reactive astrocytes also release many growth factors and trophic factors, which promote neuronal survival, synaptogenesis, neurogenesis, and angiogenesis after brain injury (Horner and Gage, 2000; Panickar and Norenberg, 2005; Shibuya, 2009). Similar to the regional specific astroglial death patterns, we have reported that reactive astrogliosis in the hippocampus originates from distinct sources and different pathways (Kim et al., 2008). One is gliogenesis in the subgranular zone of the dentate gyrus and the stratum oriens of the CA1 region. Another is *in situ* proliferation in the stratum radiatum of the CA1 region. However, the mechanisms that regulate reactive astrogliosis are complex and remain elusive.

Extracellular signal receptor-activated kinases 1/2 (ERK1/2) are members of the mitogen activated protein (MAP) kinase family, which modulates the cell cycle re-entry and cell survival (Brunet et al., 1999; Ramos et al., 2000; Formstecher et al., 2001). Nuclear ERK1/2 import triggers the entrance into the cell cycle that depends on a sustained phospho (p)-ERK1/2 nuclear accumulation (Brunet et al., 1999; Ramos et al., 2000; Formstecher et al., 2001). Indeed, ERK1/2 inhibition abrogates astroglial proliferation and up-regulation of glial fibrillary acidic protein (GFAP) expression, which are hallmarks of reactive astrogliosis (Meini et al., 2008; Sticozzi et al., 2013). However, ERK1/2 activation also alters astroglial vulnerability to oxidative stress (Regan et al., 2008). Therefore, the roles of ERK1/2 signaling pathway in astroglial death and reactive astrogliosis are still unknown.

Leptomycin B (LMB) is an anti-fungal agent and inhibits chromosomal maintenance 1 (CRM1)/exportin-dependent nuclear export. Furthermore, LMB has a potent anti-inflammatory (Loewe et al., 2002) and anti-proliferative (Lu et al., 2012) properties. Therefore, it is plausible that LMB would inhibit reactive astrogliosis. However, LMB increases ERK1/2 activity mediated by protein kinase A (PKA) and protein phosphatase 2B (PP2B, calcineurin) phosphorylations (Min et al., 2017), although ERK1/2 activation results in elevated CRM1 level in cortical astrocytes (Hayakawa et al., 2010). These contradictory observations are difficult to reconcile with a direct and general role of the ERK1/2 pathway in cell proliferation and suggest that other signaling molecules beyond ERK1/2 may be also involved in reactive astrogliosis.

p27^Kip1^, an endogenous cyclin-dependent kinase (CDK) inhibitor, acts as a primary negative regulator of cell proliferation in a variety of cell types. In quiescent cells, p27^Kip1^ is localized in the nucleus and can be transported to cytoplasm via CRM1 in response to various stimuli. Furthermore, ERK1/2 signaling pathway down-regulates nuclear p27^Kip1^ level (Toyoshima and Hunter, 1994; Gysin et al., 2005; Sakakibara et al., 2005; Zhang et al., 2010). Considering LMB-induced ERK1/2 activation (Min et al., 2017) and ERK1/2-mediated up-regulation of CRM1 in astrocytes (Hayakawa et al., 2010), the relationship between ERK1/2 activation and nucleocytoplasmic p27^Kip1^ export in reactive astrogliosis is worthy to be elucidated.

Here, we demonstrate that SE led to reactive astrogliosis showing the increases in GFAP expression and the number of proliferating (Ki-67 positive) astrocytes with prolonged ERK1/2 activation (phosphorylation) and reduced nuclear p27^Kip1^ level. U0126, an ERK1/2 inhibitor, reversed these phenomena. In contrast, LMB prevented nucleocytoplasmic p27^Kip1^ export and astroglial proliferation, although it up-regulated ERK1/2 phosphorylation and GFAP expression. Furthermore, roscovitine ameliorated the reduced nuclear p27^Kip1^ level and astroglial proliferation without changing GFAP expression and ERK1/2 phosphorylation. Therefore, these findings indicate that nucleocytoplasmic p27^Kip1^ export may be required for ERK1/2-mediated astroglial proliferation during reactive astrogliosis.

## Materials and Methods

### Experimental Animals and Chemicals

Adult male Sprague-Dawley (SD) rats (weight 250–280 g, Daehan Biolink, South Korea) were used in the study. Animals were kept under controlled environmental conditions (23–25°C, 12 h light/dark cycle) with free access to water and standard laboratory food. All animal experiments were approved by the Institutional Animal Care and Use Committee of the Hallym University (Chuncheon, South Korea). All reagents were obtained from Sigma-Aldrich (St. Louis, MO, USA), unless otherwise noted.

### SE Induction

SE was induced by a single dose (380 mg/kg) of pilocarpine in rats, as previously described (Hyun et al., 2016). Before pilocarpine injection, animals were given methylscopolamine (5 mg/kg i.p.) to block the peripheral effect of pilocarpine. Two hours after SE, animals received diazepam (10 mg/kg, i.p.) to terminate SE. As controls, rats were treated with saline instead of pilocarpine.

### Intracerebroventricular Drug Infusion

Next day, animals were stereotaxically implanted a brain infusion kit 1 (Alzet, Cupertino, CA, USA) into the right lateral ventricle (1 mm posterior; 1.5 mm lateral; −3.5 mm depth; flat skull position with bregma as reference) under Isoflurane anesthesia (1%–2% in O_2_ and N_2_O). Thereafter, an infusion kit was connected to an osmotic pump (1007D, Alzet, Cupertino, CA, USA) containing: (1) vehicle, (2) U0126 (a selective ERK1/2 inhibitor, 25 μM; Ko and Kang, 2017), (3) LMB (30 mg/ml; Min et al., 2017) or (4) roscovitine (25 μM). In addition, some control animals were given roscovitine (25 or 100 μM) by the same method.

### Tissue Processing

Three and 7 days after SE, animals were deeply anesthetized with urethane anesthesia (1.5 g/kg, i.p.) and perfused with phosphate-buffered saline (PBS, pH 7.4) followed by 4% paraformaldehyde in 0.1 M phosphate buffer (PB, pH 7.4). The brains were removed, and cryoprotected by infiltration with 30% sucrose overnight. Thereafter, the tissues were sectioned with a cryostat at 30 μm and consecutive sections were collected in six-well plates containing PBS. For western blot study, the hippocampus was rapidly removed and homogenized in lysis buffer. To analyze nuclear p27^Kip1^ level, the stratum radiatum of the CA1 region were rapidly dissected, homogenized and fractionated with Subcellular Protein Fractionation Kit for Tissues (Thermo Scientific, Waltham, MA, USA), according to the manufacturer’s instructions. The protein concentration in the supernatant was determined using a Micro BCA Protein Assay Kit (Pierce Chemical, Dallas, TX, USA).

### Fluoro-Jade B (FJB) and TUNEL Staining

Fluoro-Jade B (FJB) staining was performed as previously described (Hyun et al., 2016). Briefly, brain sections were rinsed in distilled water, and mounted onto gelatin-coated slides. Slides were immersed in ethanol and distilled water. The slides were then transferred to 0.06% potassium permanganate. After rinsing in distilled water, the slides were incubated for 30 min in 0.001% FJB (Histo-Chem Inc., Jefferson, AR, USA). TUNEL staining was performed with the ApopTag^®^ Fluorescein *In Situ* Apoptosis Detection Kit (Millipore, S7110, USA) according to the manufacturer’s protocol^1^. Following the TUNEL reaction, double fluorescent staining was performed (see below).

### Immunohistochemistry

Free-floating sections were first incubated with 10% normal goat serum (Vector, Burlingame, CA, USA), and then reacted with mouse anti-GFAP antibody (1:1000, Millipore, #MAB3402, USA, diluted 1:1000) in PBS containing 0.3% Triton X-100 and 2% normal goat serum overnight at room temperature. After washing, sections were incubated sequentially, in horse anti-mouse IgG (Vector, Burlingame, CA, USA) and ABC complex (Vector, Burlingame, CA, USA). Between the incubations, the tissues were washed with PBS three times. The sections were visualized with 3,3′-diaminobenzidine (DAB) in 0.1 M Tris buffer and mounted on the gelatin-coated slides. Some sections were incubated in a mixture of rabbit anti-GFAP (1:100, Millipore, AB5804, USA)/mouse anti-Ki-67 (1:100, Novocastra Laboratories, NCL-Ki67-MM1, UK) or rabbit anti-p27^Kip1^ (1:100, Abcam, ab7961, UK)/mouse anti-GFAP (1:200, Millipore, #MAB3402, USA) antisera in PBS containing 0.3% Triton X-100 overnight at room temperature. After washing three times for 10 min with PBS, the sections were also incubated in a mixture of FITC- and Cy3-conjugated secondary antisera (or streptavidin, 1:250, Amersham, USA) for 2 h at room temperature. The sections were washed and mounted on gelatin-coated slides. All images were captured using an Axio Imager M2 microscope and AxioVision Rel. 4.8 software (Carl Zeiss Korea, Seoul, South Korea). For quantitative analysis, images of the CA1 region and DG were captured (15 sections per each animal), and areas of interest (1 × 10^5^ μm^2^) were selected. Thereafter, two different investigators performed FJB or TUNEL- or Ki-67 positive cell counts and measured p27^Kip1^ fluorescent intensity using AxioVision Rel. 4.8 software.

### Western Blot

Western blotting was performed according to standard procedures. Briefly, Tissue lysate proteins were blotted onto nitrocellulose transfer membranes (Schleicher and Schuell BioScience Inc., Keene, NH, USA), then incubated with mouse anti-GFAP (1:5000, Millipore, #MAB3402, USA), rabbit anti-ERK1/2 (1:1000, Biorbyt, orb160960, USA), rabbit anti-pERK1/2 (1:1000, Millipore, #05-797RSP, USA) or rabbit anti-p27^Kip1^ (1:1000, Abcam, ab7961, UK) antibody. Immunoreactive bands were detected and quantified on ImageQuant LAS4000 system (GE Healthcare, Chicago, IL, USA). The rabbit anti-β-actin (for total extracts, 1:6000, Sigma, USA) or rabbit anti-poly(ADP-ribose) polymerase-1 (PARP1, for nuclear fraction; 1:500, Abnova, USA) primary antibody was used as internal reference.

### Statistical Analysis

All data obtained from the quantitative measurements were tested for the normality and equality of variance. Thereafter, data were analyzed by one-way analysis of variance (ANOVA) coupled with Bonferroni’s *post hoc* test for multiple comparison. Values are presented as mean ± SEM. Differences were considered as significant for *p* < 0.05.

## Results

### Effects of U0126 and LMB on GFAP Expression and ERK1/2 Phosphorylation Following SE

**Figure 1:**
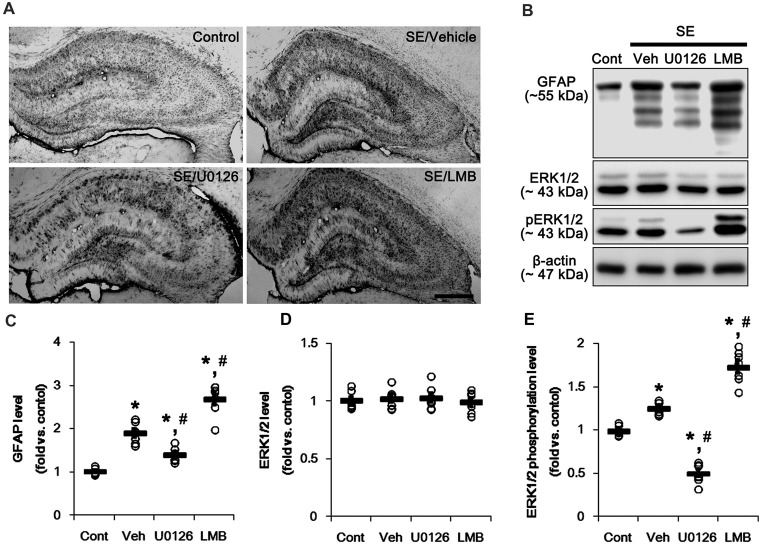
Effects of U0126 and leptomycin B (LMB) on glial fibrillary acidic protein (GFAP), extracellular signal receptor activatedkinases 1/2 (ERK1/2) and phospho (p)-ERK1/2 level in the hippocampus following status epilepticus (SE). **(A)** Representative images for GFAP expression in the hippocampal tissues. As compared to vehicle, U0126 increases GFAP expression, while LMB reduces it. Bar = 400 μm. **(B)** Representative images for western blot images of GFAP, ERK1/2 and pERK1/2 levels in the hippocampal tissues. **(C–E)** Quantifications of GFAP **(C)**, ERK1/2 **(D)** and pERK1/2 **(E)** intensity. Open circles indicate each individual value. Horizontal bars indicate mean value. Error bars indicate SEM (*,^#^*p* < 0.05 vs. control and vehicle, respectively;* n* = 7, respectively).

First, we investigated the effects of U0126 and LMB on GFAP expression and ERK1/2 phosphorylation in the hippocampus 7 days after SE. Consistent with our previous studies (Kang et al., 2006; Kim et al., 2008, 2017), GFAP expression showed the spatio-temporal specific pattern in the hippocampus following SE. Briefly, GFAP expression was markedly elevated in the stratum radiatum of the CA1 field (Figure 1A). In contrast, the GFAP-deleted area was observed in the molecular layer of the dentate gyrus (Figure 1A). As compared to vehicle, U0126 abolished the up-regulation of GFAP expression induced by SE, while LMB enhanced it (Figure 1A). However, both U0126 and LMB did not affect the distinct GFAP expression pattern in the hippocampus following SE.

**Figure 2:**
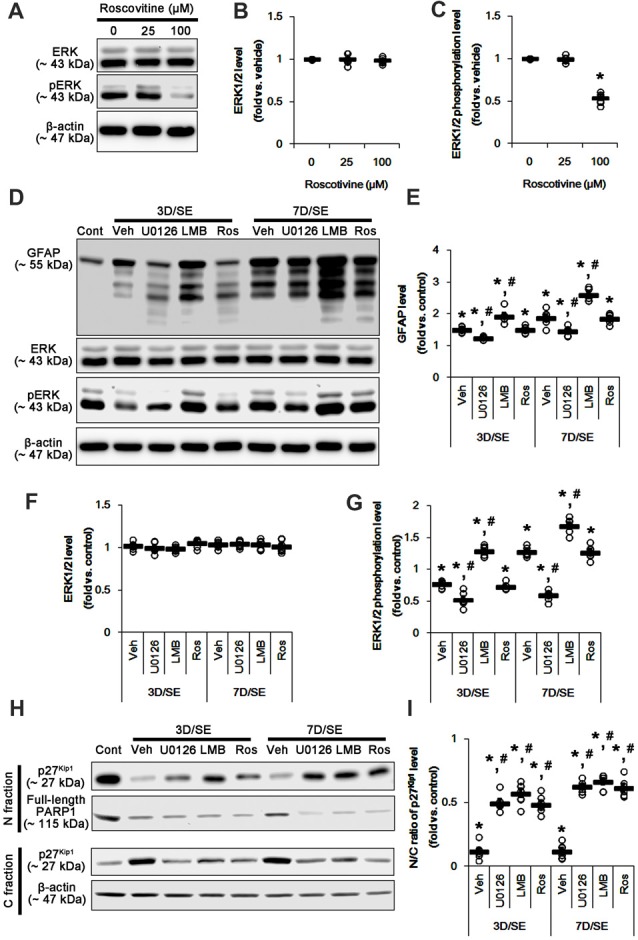
Effects of U0126, LMB and roscovitine on GFAP, ERK1/2, phospho (p)-ERK1/2 and p27^Kip1^ levels following SE. **(A–C)** Effect of roscovitine on ERK1/2 phosphorylation in control animals. **(A)** Representative images for western blot of ERK1/2 and pERK level expression in the hippocampal tissues. **(B,C)** Quantifications of EKR1/2 **(B)** and pERK1/2 **(C)** intensity. Open circles indicate each individual value. Horizontal bars indicate mean value. Error bars indicate SEM (*,^#^*p* < 0.05 vs. control and vehicle, respectively;* n* = 7, respectively). **(D–G)** Effects of U0126, LMB and roscovitine on GFAP, ERK1/2 and pERK1/2 levels 3-day and 7-day post-SE animals. **(D)** Representative images for western blot of GFAP, ERK1/2 and pERK1/2 level in the hippocampal tissues. **(E–G)** Quantifications of GFAP **(C)** ERK1/2 **(D)** and pERK1/2 **(E)** intensity. Open circles indicate each individual value. Horizontal bars indicate mean value. Error bars indicate SEM (*,^#^*p* < 0.05 vs. control and vehicle, respectively;* n* = 7, respectively). **(H,I)** Effects of U0126, LMB and roscovitine on subcellular p27^Kip1^ level 3-day and 7-day post-SE animals. **(H)** Representative images for western blot of p27^Kip1^ level in nuclear (N) and cytosolic (S) fractions. **(I)** Quantifications of the ratio of p27^Kip1^ level in nuclear and cytosolic fractions. Open circles indicate each individual value. Horizontal bars indicate mean value. Error bars indicate SEM (*,^#^*p* < 0.05 vs. control and vehicle, respectively;* n* = 7, respectively).

Western blots revealed that GFAP expression and ERK1/2 phosphorylation were increased in the whole hippocampus 7 days after SE (*p* < 0.05 vs. vehicle, respectively; Figures 1B,C and Supplementary Figure S1). U0126 ameliorated the up-regulated GFAP expression and ERK1/2 phosphorylation induced by SE, while LMB showed opposite effects (*p* < 0.05 vs. vehicle, respectively; Figures 1B,C and Supplementary Figure S1). To explore the chronological effects of U0126 and LMB on reactive astrogliosis, we also investigated GFAP expression and ERK1/2 phosphorylation in 3-day post-SE groups. As compared to control animals, U0126 attenuated GFAP expression accompanied by the reduced ERK1/2 phosphorylation at 3 days after SE (*p* < 0.05 vs. vehicle; Figures 2D–G and Supplementary Figure S2). LMB also showed completely opposite effects on GFAP expression and ERK1/2 phosphorylation at this time window (*p* < 0.05 vs. vehicle; Figures 2D–G and Supplementary Figure S2). With respect to our previous reports demonstrating the effects of U0126 and LMB on ERK1/2 phosphorylation (Ko and Kang, 2017; Min et al., 2017), these findings indicate that ERK1/2 phosphorylation (activation) may play an important role in the up-regulation of GFAP expression in reactive astrocytes following SE.

### Effects of U0126, LMB and Roscovitine on Nuclear p27^Kip1^ Level Following SE

Reactive astrogliosis refers to astroglial proliferation accompanied by the up-regulated GFAP expression (Ridet et al., 1997; Kim et al., 2008). p27^Kip1^ is a primary negative regulator of cell proliferation (Toyoshima and Hunter, 1994; Zhang et al., 2010). CRM1-mediated nucleocytoplasmic p27^Kip1^ export and/or its degradation lead to re-enter the cell cycle in various cells including neurons (Toyoshima and Hunter, 1994; Kim et al., 2014b). Thus, we validated whether U0126 or LMB affects nucleocytoplasmic p27^Kip1^ export induced by SE. In the present study, SE significantly reduced the ratio of p27^Kip1^ level in the nuclear and cytosolic fractions obtained from the stratum radiatum of the CA1 region (*p* < 0.05 vs. control; Figures 2H,I and Supplementary Figure S3) where astrocytes were abundantly localized (see Figure 1A). U0126 and LMB attenuated the SE-induced reduction in the ratio of p27^Kip1^ level in the nuclear and cytosolic fractions at 3 and 7 days after SE (*p* < 0.05 vs. vehicle; Figures 2H,I and Supplementary Figure S3). Consistent with our previous study (Kim et al., 2014a), SE led to PARP1 degradation in every groups. Regardless of the underlying mechanisms of PARP1 degradation in the nuclear fraction, our findings reveal that U0126- and LMB-mediated inhibition of nucleocytoplamic p27^Kip1^ export may prevent reactive astrogliosis.

To further elucidate the role of p27^Kip1^ in reactive astrogliosis, we applied roscovitine to rats prior to SE induction. This is because roscovitine, a cyclin-dependent kinase 5 (CDK5) inhibitor, is a poor ERK1/2 inhibitor with IC_50_ values of 34 μM and 14 μM for ERK1 and ERK2, respectively (Meijer et al., 1997), but effectively increases nuclear p27^Kip1^ expression/accumulation independent of CRM1 activity (Foster et al., 2003; Zhang et al., 2004). Furthermore, roscovitine attenuates astroglial proliferation (Di Giovanni et al., 2005). Indeed, we have reported that 100 μM roscovitine prevents reactive astrogliosis via PKA dependent pathway following SE (Hyun et al., 2017). Therefore, it is likely that roscovitine may be an ideal compound to regulate nuclear p27^Kip1^ level without affecting CRM1 and ERK1/2 activities.

Based on these previous studies, we first investigated the optimal dose of roscovitine to increase nuclear p27^Kip1^ level without affecting ERK1/2 phosphorylation in control animals. As compared to vehicle, 25 μM roscovitine did not influence ERK1/2 phosphorylation, while 100 μM roscovitine reduced it (Figures 2A–C and Supplementary Figure S2). Thus, we used 25 μM roscovitine in the present study. Following SE, 25 μM roscovitine did not affect the alterations in GFAP expression and ERK1/2 phosphorylation, as compared to vehicle (Figures 2D–G and Supplementary Figure S2). However, roscotivine abolished the SE-induced reduction in the ratio of p27^Kip1^ level in the nuclear and cytosolic fractions at 3 and 7 days after SE (*p* < 0.05 vs. vehicle; Figures 2H,I and Supplementary Figure S3). Therefore, these findings indicate that the prevention of the reduced nuclear p27^Kip1^ level may inhibit reactive astrogliosis, independent of ERK1/2 and CRM1 activities.

### Effects of U0126, LMB and Roscovitine on Nucleocytoplasmic p27^Kip1^ Export Following SE

**Figure 3:**
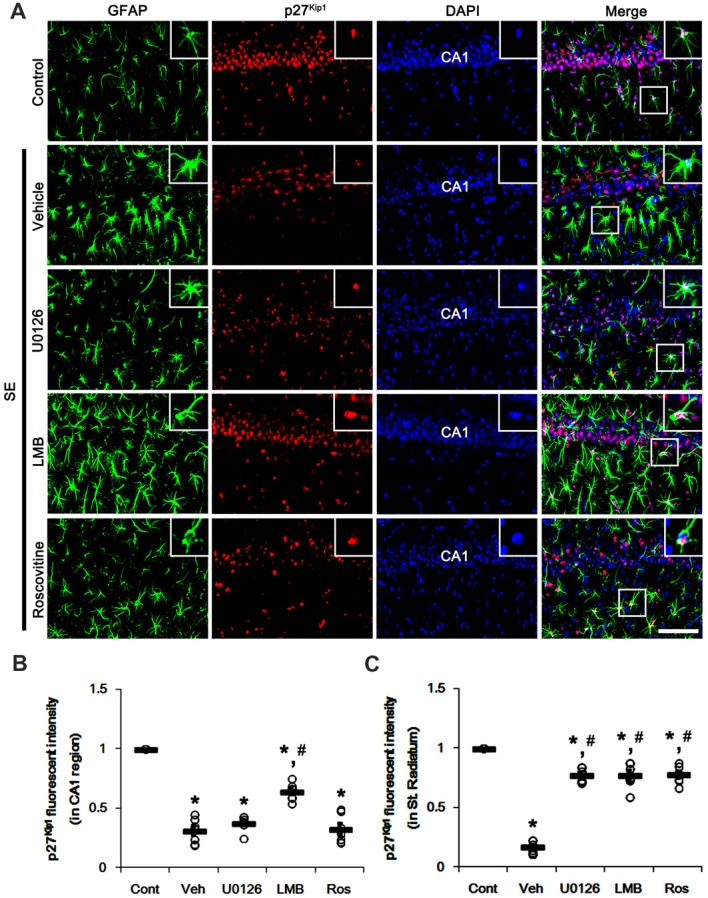
Effects of U0126, LMB and roscovitine on p27^Kip1^ expression in the CA1 region following SE. **(A)** Representative double immunofluorescent images for GFAP and p27^Kip1^ expression. SE reduces nuclear p27^Kip1^ expression in CA1 neurons as well as astrocytes. As compared to vehicle, LMB attenuates the decline of nuclear p27^Kip1^ expression in CA1 neurons, while U0126 and roscovitine do not. However, U0126, LMB and roscovitine abrogate the decline of nuclear p27^Kip1^ expression in astrocytes. Insert images are high magnification of rectangles in merge panels. Bar = 50 and 12.5 (inserts) μm. **(B,C)** Quantifications of the number of nuclear p27^Kip1^ expression in CA1 neuron **(B)** and astrocytes **(C)**. Open circles indicate each individual value. Horizontal bars indicate mean value. Error bars indicate SEM (*,^#^*p* < 0.05 vs. control and vehicle, respectively;* n* = 7, respectively).

To confirm the role of nuclear p27^Kip1^ level in reactive astrocytes, we investigated whether U0126, LMB and roscovitine actually prevent nucleocytoplasmic p27^Kip1^ export in astrocytes following SE. As compared to control animals, SE significantly reduced nuclear p27^Kip1^ levels in CA1 neurons and CA1 astrocytes (*p* < 0.05 vs. control animals; Figures 3A–C). U0126 and roscovitine effectively attenuated the decreased nuclear p27^Kip1^ levels in CA1 astrocytes, not CA1 neurons, induced by SE (*p* < 0.05 vs. vehicle; Figures 3A–C). In contrast, LMB inhibited the SE-induced decline of nuclear p27^Kip1^ levels in CA1 neurons and CA1 astrocytes (*p* < 0.05 vs. vehicle; Figures 3A–C).

**Figure 4:**
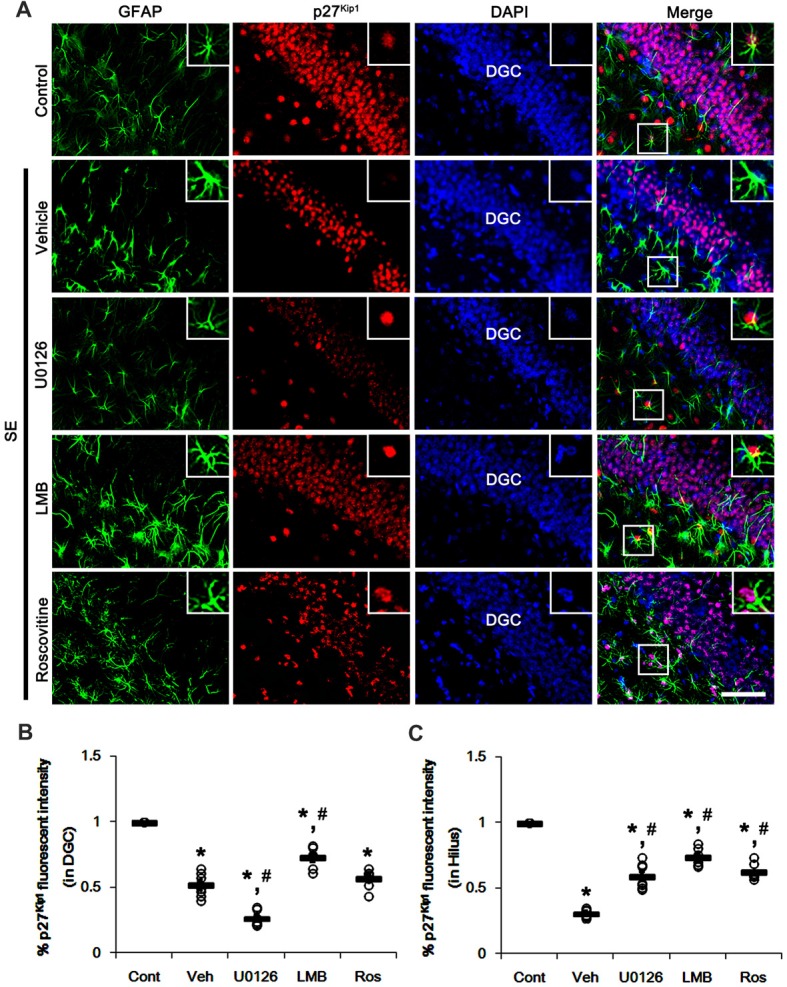
Effects of U0126, LMB and roscovitine on p27^Kip1^ expression in the dentate gyrus following SE. **(A)** Representative double immunofluorescent images for GFAP and p27^Kip1^ expression. SE reduces nuclear p27^Kip1^ expression in dentate granule cells (DGC) as well as astrocytes. As compared to vehicle, U0126 aggravates the decline of nuclear p27^Kip1^ expression in DGC, while LMB attenuates it. Roscovitine does not affect SE-induced reduction in nuclear p27^Kip1^ expression in DGC. However, three distinct compounds mitigate the decline of nuclear p27^Kip1^ expression in astrocytes. Insert images are high magnification of rectangles in merge panels. Bar = 50 and 12.5 (inserts) μm. **(B,C)** Quantifications of the number of nuclear p27^Kip1^ expression in DGC **(B)** and astrocytes **(C)**. Open circles indicate each individual value. Horizontal bars indicate mean value. Error bars indicate SEM (*,^#^*p* < 0.05 vs. control and vehicle, respectively;* n* = 7, respectively).

Similar to CA1 neurons, dentate granule cells (DGC) showed the reduction in nuclear p27^Kip1^ level following SE (*p* < 0.05 vs. control animals; Figures 4A–C). As compared to vehicle, U0126 exacerbated the down-regulation of nuclear p27^Kip1^ level in DGC (*p* < 0.05 vs. vehicle; Figures 4A–C), while U0126 prevented the reduced nuclear p27^Kip1^ levels in astrocytes within the dentate gyrus (*p* < 0.05 vs. vehicle; Figures 4A–C). LMB ameliorated the decrease in nuclear p27^Kip1^ levels in DGC and astrocytes in the dentate gyrus (*p* < 0.05 vs. vehicle; Figures 4A–C). Roscovitine attenuated the decrease in nuclear p27^Kip1^ levels in astrocytes in the dentate gyrus (*p* < 0.05 vs. vehicle; Figures 4A–C) but did not affect p27^Kip1^ levels in DGC. Together with western blot data (Figure 2), our findings indicate that nuclear p27^Kip1^ accumulation may play an inhibitory role in reactive astrogliosis independent of ERK1/2 activity.

### Effects of U0126, LMB and Roscovitine on Astroglial Proliferation Following SE

**Figure 5:**
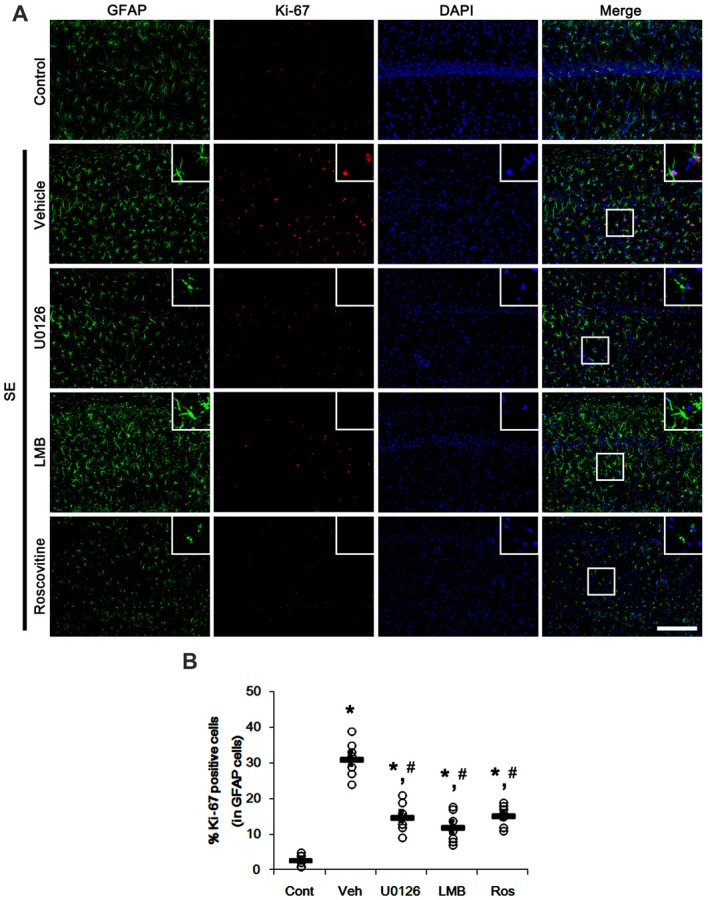
Effects of U0126, LMB and roscovitine on Ki-67 expression in the CA1 region following SE. **(A)** Representative double immunofluorescent images for Ki-67 positive astrocytes. SE increases nuclear Ki-67 expression in astrocytes. As compared to vehicle, U0126, LMB and roscovitine reduce the number of Ki-67 positive astrocytes following SE. Insert images are high magnification of rectangles in merge panels. Bar = 100 and 25 (inserts) μm. **(B)** Quantifications of the number of Ki-67 positive astrocytes. Open circles indicate each individual value. Horizontal bars indicate mean value. Error bars indicate SEM (*,^#^*p* < 0.05 vs. control and vehicle, respectively;* n* = 7, respectively).

**Figure 6:**
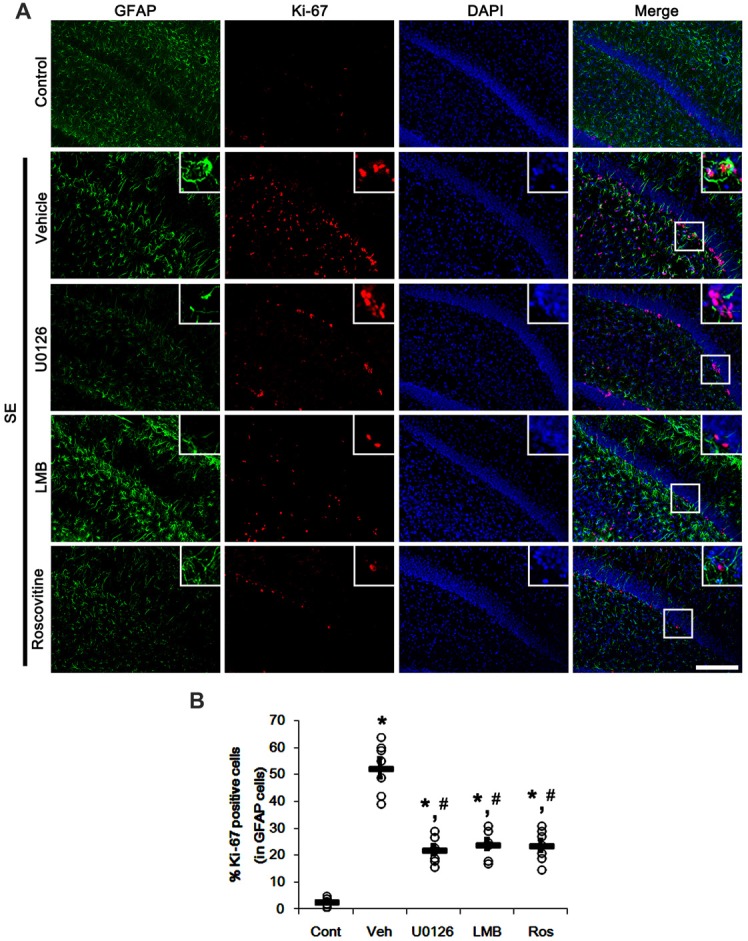
Effects of U0126, LMB and roscovitine on Ki-67 expression in the dentate gyrus following SE. **(A)** Representative double immunofluorescent images for Ki-67 positive astrocytes. SE increases nuclear Ki-67 expression in astrocytes within the subgranular zone and the hilus. As compared to vehicle, three distinct compounds reduce the number of Ki-67 positive astrocytes. Insert images are high magnification of rectangles in merge panels. Bar = 100 and 25 (inserts) μm. **(B)** Quantifications of the number of Ki-67 positive astrocytes. Open circles indicate each individual value. Horizontal bars indicate mean value. Error bars indicate SEM (*,^#^*p*< 0.05 vs. control and vehicle, respectively;* n* = 7, respectively).

Since U0126, LMB and roscovitine ameliorated the reduced nuclear p27^Kip1^ level in astrocytes (Figures 3, 4), we explored the effects of these compounds on astroglial proliferation during reactive astrogliosis induced by SE. As compared to control animals, SE increased the number of Ki-67 (a proliferative cell marker) positive astrocytes in the CA1 region (*p* < 0.05 vs. control animals; Figures 5A,B). U0126, LMB and roscovitine effectively abrogated the astroglial proliferation in the CA1 region induced by SE (*p* < 0.05 vs. vehicle; Figures 5A,B). Similar to the CA1 region, SE significantly increased the number of Ki-67 positive astrocytes in the subgranular zone and the hilar region of the dentate gyrus (*p* < 0.05 vs. control animals; Figures 6A,B). U0126, LMB and roscovitine ameliorated the Ki-67 expression in the astrocytes within these regions (*p* < 0.05 vs. vehicle; Figures 6A,B). Considering the distinct effects of U0126, LMB and roscovitine on ERK1/2 phosphorylation (Figures 1, 2), our findings indicate that ERK1/2 activation without nucleocytoplasmic p27^Kip1^ export may not be sufficient to induce astroglial proliferation during the process of reactive astrogliosis.

### Effects of U0126, LMB and Roscovitine on SE-Induced Astroglial Apoptosis and Neuronal Death

**Figure 7:**
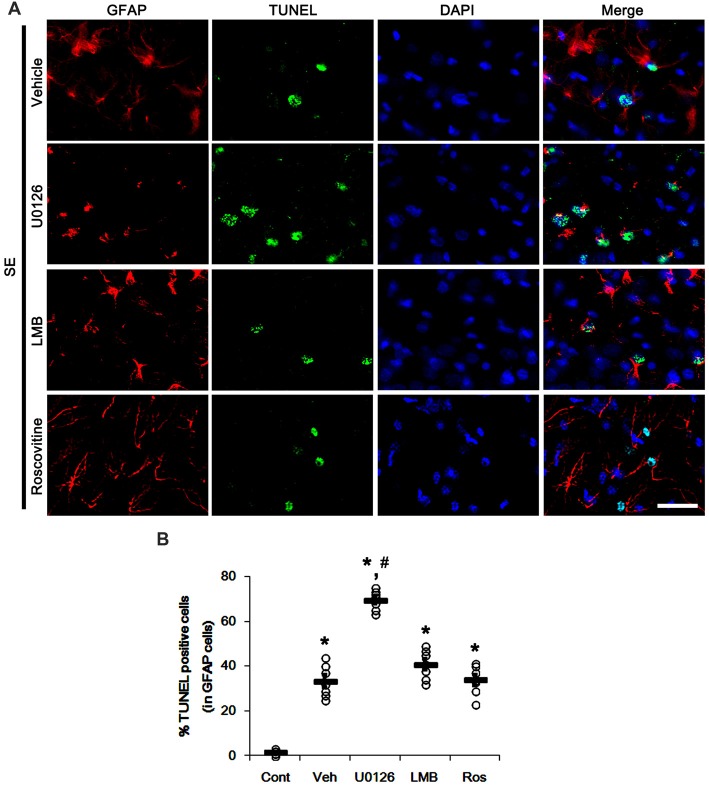
Effects of U0126, LMB and roscovitine on astroglial apoptosis in the molecular layer of the dentate gyrus following SE. **(A)** Representative photos demonstrating TUNEL-positive astrocytes. As compared to vehicle, U0126 exacerbates SE-induced astroglial apoptosis, while LMB and roscovitine do not. Bar = 12.5 μm. **(B)** Quantification of the fractions of TUNEL-positive astrocytes in total astrocytes within the molecular layer of the dentate gyrus. Open circles indicate each individual value. Horizontal bars indicate mean value. Error bars indicate SEM (*,^#^*p* < 0.05 vs. control and vehicle, respectively;* n* = 7, respectively).

We have reported that SE results in the massive astroglial apoptosis in the molecular layer of the dentate gyrus and programmed CA1 neuronal necrosis (Kang et al., 2006; Kim et al., 2014a,b; Ko et al., 2015). More recently, we have also reported that U0126 facilitates SE-induced neuronal death, but LMB attenuates it (Hyun et al., 2016; Ko and Kang, 2017; Min et al., 2017). With respect to these previous studies, it is likely that U0126, LMB and roscovitine would indirectly affect GFAP expression and astroglial proliferation by regulating astroglial apoptosis and neuronal death following SE. Therefore, we evaluated the effects of U0126, LMB and roscovitine on SE-induced astroglial apoptosis and neuronal damage. Following SE, TUNEL-positive astrocytes were obviously detected in the molecular layer of the dentate gyrus (*p* < 0.05 vs. control animals; Figures 7A,B). U0126 aggravated SE-induced astroglial apoptosis in this region (*p* < 0.05 vs. vehicle; Figures 7A,B) that was unaffected by LMB and roscovitine (Figures 7A,B).

**Figure 8:**
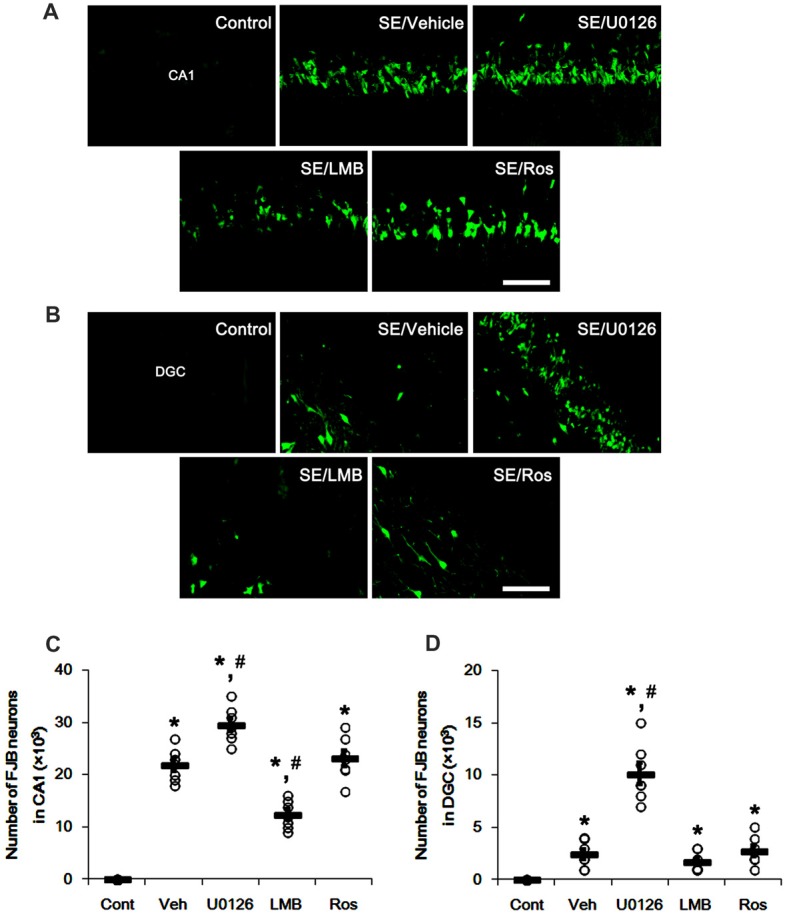
Effects of U0126, LMB and roscovitine on neuronal damage in the CA1 region and the dentate gyrus. **(A,B)** Representative images for Fluoro-Jade B (FJB) signals in CA1 neurons **(A)** and DGC **(B)** Bar = 50 μm. As compared to vehicle, U0126 deteriorates SE-induced neuronal damage in CA1 neurons and DGC, while LMB attenuates neuronal damage only in CA1 neurons. Roscovitine does not affect SE-induced neuronal death in CA1 neurons and DGC. **(C,D)** Quantification of the number of FJB-positive CA1 neurons **(C)** and DGC **(D)** Open circles indicate each individual value. Horizontal bars indicate mean value. Error bars indicate SEM (*,^#^*p* < 0.05 vs. control and vehicle, respectively;* n* = 7, respectively).

SE also resulted in massive neuronal death in CA1 neurons, but not DGC (*p* < 0.05 vs. control animals; Figures 8A–D). U0126 exacerbated SE-induced CA1 neuronal death and evoked DGC degeneration, as compared to vehicle (*p* < 0.05 vs. vehicle; Figures 8A–D). LMB mitigated SE-induced neuronal death in the CA1 region (*p* < 0.05 vs. vehicle; Figures 8A–D) and did not induce DGC degeneration. Roscovitine did not affect SE-induced CA1 or DGC degeneration (*p* < 0.05 vs. vehicle; Figures 8A–D). Therefore, our findings indicate that the effects of U0126, LMB and roscovitine on astroglial proliferation during reactive astrogliosis may not be relevant to astroglial and neuronal degeneration induced by SE.

## Discussion

The major findings in the present study are that the release of nuclear p27^Kip1^ entrapment may be required for ERK1/2-mediated astroglial proliferation during reactive astrogliosis, although ERK1/2 activation may play an important role in up-regulation of GFAP expression in reactive astrocytes.

Reactive astrogliosis is a prominent and ubiquitous reaction of astrocytes to many types of brain injury, which is relevant to the poor regenerative capacity of the adult brain (Mandell et al., 2001). After injury, ERK1/2 activation induces up-regulation of GFAP expression and triggers astroglial proliferation, which are hallmarks of reactive astrogliosis (Meini et al., 2008; Sticozzi et al., 2013). In the present study, we found that GFAP expression and ERK1/2 phosphorylation were increased in the rat hippocampus following SE. In addition, U0126 abrogated the up-regulation of GFAP expression and the number of proliferative astrocytes in the CA1 region. These findings are consistent with previous studies demonstrating anti-astrogliotic effects of ERK1/2 inhibitors (Sorensen et al., 2003; Meini et al., 2008; Sticozzi et al., 2013). Unexpectedly, we also found that LMB effectively inhibited astroglial proliferation, although it up-regulated GFAP expression and ERK1/2 phosphorylation following SE. Furthermore, roscovitine abrogated astroglial proliferation without changing GFAP expression and ERK1/2 phosphorylation induced by SE. These findings indicate that ERK1/2 activation may not be sufficient to induce astroglial proliferation during reactive astrogliosis, although it may be one of the regulatory molecules in up-regulation of GFAP expression.

p27^Kip1^ is one of the primary negative regulators of cell proliferation in a variety of cell types. p27^Kip1^ level is decreased in response to mitogenic stimulations. Furthermore, up-regulated p27^Kip1^ protein level causes cells to arrest in the G1 phase of the cell cycle (Connor et al., 2003; Alkarain et al., 2004). Interestingly, ERK1/2 signaling pathway regulates the mitogen-induced down-regulation of p27^Kip1^ (Meloche and Pouysségur, 2007). In the present study, U0126 resulted in nuclear p27^Kip1^ entrapment in astrocytes accompanied by the reduced ERK1/2 phosphorylation following SE. However, LMB decreased SE-induced nucleocytoplasmic p27^Kip1^ transport in astrocytes, in spite of ERK1/2 hyperactivation. This finding is consistent with a previous study reporting that CRM1 inhibition mediates persistent ERK1/2 hyperactivation and leads to G1 cell cycle arrest (Pathria et al., 2012). Since ERK1/2 up-regulates CRM1 expression in astrocytes (Hayakawa et al., 2010), it is likely that LMB-induced ERK1/2 hyperactivation may be one of the compensatory responses for CRM1 inhibition. Furthermore, LMB influences expression levels and activities of nuclear factor-κB, signal transducer and activator of transcription 3, p53, p27, p21, survivin and cytokines, which are involved in reactive astrogliosis and apoptosis (Zhai et al., 2004; Beurel and Jope, 2008; El-Hage et al., 2008; Turner and Sullivan, 2008; Pathria et al., 2012). Therefore, it is not excluded that the alteration of the nuclear export of other CRM1-targets, not p27^Kip1^, would also inhibit reactive astrogliosis. Further studies are needed to elucidate the regulatory mechanisms of ERK1/2 activity by LMB. In the present study, roscovitine also preserved nuclear p27^Kip1^ level without altered ERK1/2 phosphorylation. Furthermore, these nuclear p27^Kip1^ entrapments by three different compounds were concomitant with the reduced number of Ki-67 positive proliferative astrocytes. Therefore, our findings suggest that nucleocytoplamic p27^Kip1^ export may be required for ERK1/2-mediated astroglial proliferation during reactive astrogliosis.

Since astroglial viability in response to oxidative stress is improved by U0126 (Regan et al., 2008), ERK1/2 affects astroglial vulnerability to stress, which is involved in anti-apoptotic properties of astrocytes under pathophysiological conditions (Zhang et al., 2007). However, ERK1/2 also plays a key role in astroglial apoptosis (Kawasaki et al., 2007). In the present study, ERK1/2 inhibition by U0126 aggravated astroglial apoptosis in the molecular layer of the dentate gyrus following SE, while LMB-induced persistent ERK1/2 hyperactivation did not affect astroglial apoptosis in this region. These findings suggest that ERK1/2 may not be a decisive factor for astroglial apoptosis induced by SE.

On the other hand, we have recently reported that LMB attenuated SE-induced programmed CA1 neuronal necrosis via ERK1/2 activation (Min et al., 2017). The present study also shows that LMB ameliorated SE-induced neuronal death in the CA1 region, while U0126 aggravated SE-induced CA1 neuronal death and DGC degeneration. Consistent with our previous study (Hyun et al., 2017), roscovitine was ineffective to SE-induced neuronal death. These findings indicate that ERK1/2 activation may be more neuroprotective than nuclear p27^Kip1^ entrapment against SE insults. Otherwise, it is likely that the efficacy of LMB on p27^Kip^ entrapment and the efficiency of nucleocytoplasmic p27^Kip^ export in post-mitotic neurons may be distinct from those in proliferative astrocytes following SE. Regardless of the results concerning neuronal death and astroglial apoptosis, our findings indicate that the inhibitory effects of U0126, LMB and roscovitine on astroglial proliferation may be independent of neuronal death and astroglial apoptosis.

Recently, it has been reported that neuroinflammation and ischemia induce two different types of astroglial reactivity termed “A1” and “A2”, respectively (Zamanian et al., 2012). Since A1 reactive astrocytes secrete neurotoxic cytokines and A2 reactive astrocytes release neurotrophic factors, these distinct subpopulations of reactive astrocytes play a harmful and protective role in neurons, respectively. Furthermore, A1 reactive astrocytes are highly present in the human brains obtained from patients of neurodegenerative diseases and aged mice brains (Liddelow et al., 2017; Clarke et al., 2018). Therefore, it is likely that, beyond GFAP upregulation and astroglial proliferation, reactive astrogliosis may include the differential signaling pathways and the acquisition of alternative phenotypes depending on the type of insults. Similarly, we have reported two regional specific astroglial death patterns in the hippocampus induced by SE. One is acute astroglial apoptosis in the molecular layer, and another is clasmatodendrosis (an autophagic astroglial degeneration) in the CA1 region (Kim et al., 2008, 2010, 2011, 2014a,b, 2017). Therefore, it is likely that astrocytes may have distinct properties in region-dependent manners. In the present study, U0126 was toxic, LMB was protective, and roscovitine was ineffective to SE-induced neuronal death, although all compounds effectively inhibited astroglial proliferations in the CA1 region and the hilus of the dentate gyrus. In contrast, U0126 aggravated SE-induced astroglial apoptosis in the molecular layer of the dentate gyrus that was unaffected by LMB and roscovitine. Based on the reports concerning the distinct properties of reactive astrocytes (Zamanian et al., 2012; Liddelow et al., 2017; Clarke et al., 2018), our findings suggest that nucleocytoplasmic p27^Kip1^ transport may differently contribute to the proliferation and vulnerability of A1/A2 astrocytes in response to brain injury. Further studies are needed to elucidate the regulatory mechanisms of ERK1/2, CDK5 and p27^Kip1^ in the acquisition of alternative phenotypes of A1/A2 astrocytes.

To the best of our knowledge, the present data validate for the first time the roles of ERK1/2 and CRM1 in astroglial proliferation via nucleocytoplasmic p27^Kip1^ transport during reactive astrogliosis. Therefore, our findings suggest that nuclear p27^Kip1^ entrapment may be a potential therapeutic strategy for anti-proliferation in reactive astrocytes rather than ERK1/2 inhibition.

## Author Contributions

T-CK designed and supervised the project. J-EK and T-CK performed the experiments described in the manuscript, analyzed the data and wrote the manuscript.

## Conflict of Interest Statement

The authors declare that the research was conducted in the absence of any commercial or financial relationships that could be construed as a potential conflict of interest.

## Footnotes

^1^http://www.emdmillipore.com/US/en/product/ApopTag-Fluorescein-In-Situ-Apoptosis-Detection-Kit,MM_NF-S7110?bd=1

## Supplementary Material

The Supplementary Material for this article can be found online at: https://www.frontiersin.org/articles/10.3389/fncel.2018. 00152/full#supplementary-material
