# Supplementary material for: Nucleocytoplasmic p27Kip1 Export Is Required for ERK1/2-Mediated Reactive Astroglial Proliferation Following Status Epilepticus
Source: Front Cell Neurosci. 2018 Jun 7;12:152. doi: 10.3389/fncel.2018.00152 (PMC5999727; doi:10.3389/fncel.2018.00152)
Supplement: Supplementary file 1 [file Data_Sheet_1.PDF]

Supplementary information

Nucleocytoplasmic $p27^{Kip1}$ export is required for ERK1/2-mediated reactive astroglial proliferation following status epilepticus

Ji-Eun Kim, Tae-Cheon Kang*

Department of Anatomy and Neurobiology, Institute of Epilepsy Research, College of Medicine, Hallym University, Chuncheon 24252, South Korea.

* Correspondence should be addressed to T-C K (e-mail: tckang@hallym.ac.kr)

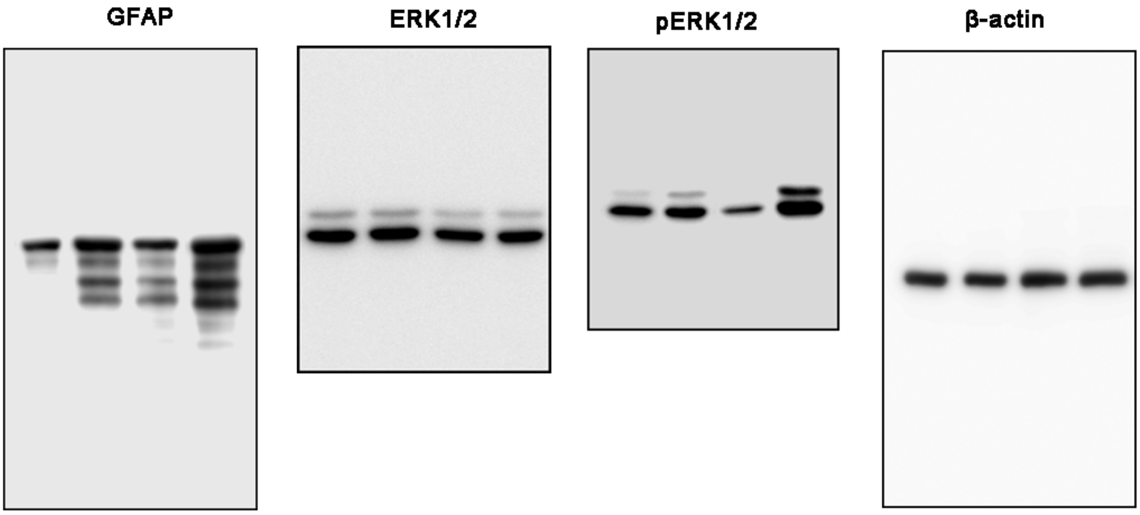

Supplementary Fig. 1. Full-length gel images of western blot data in Fig. 1.

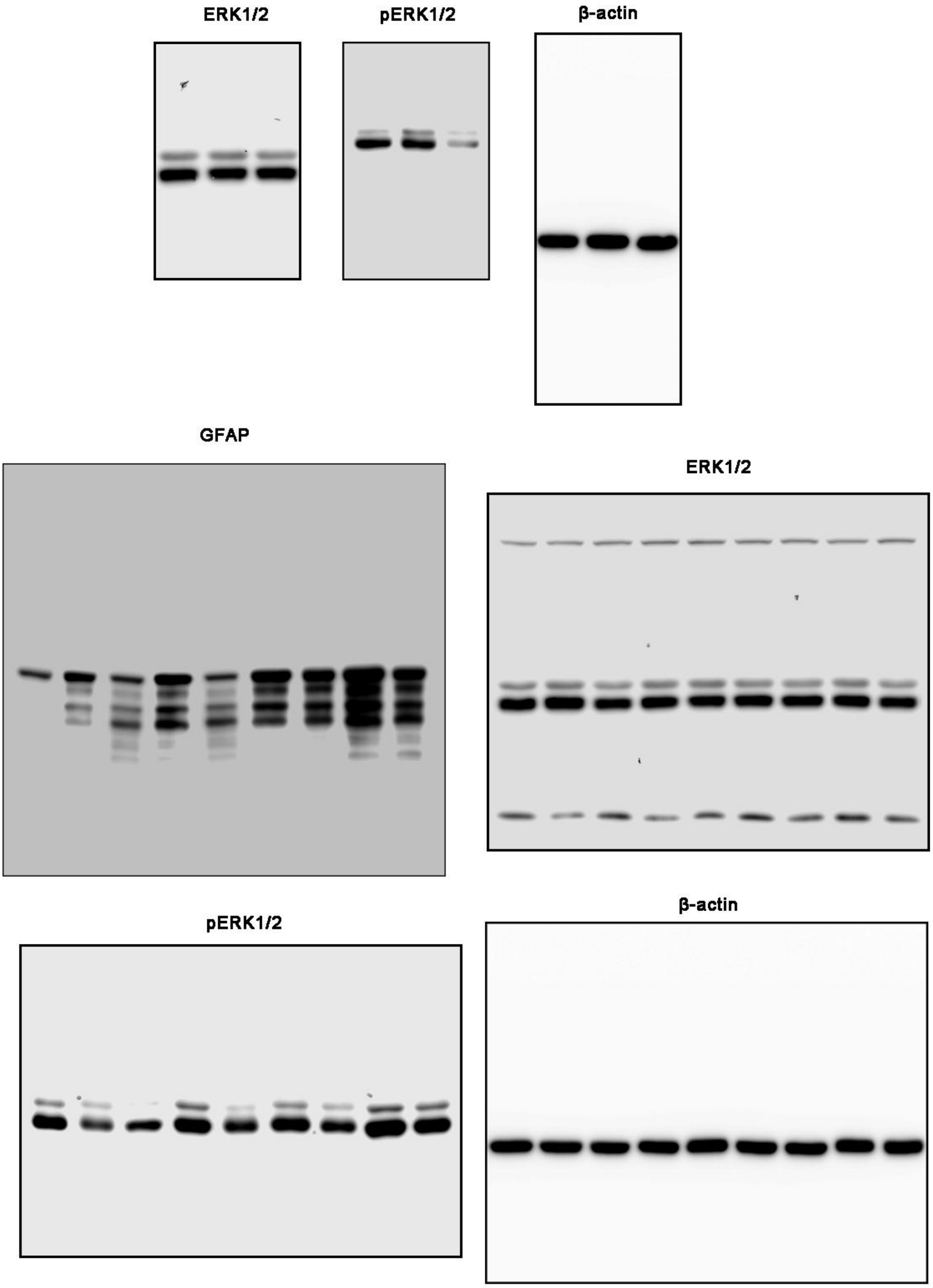

Supplementary Fig. 2. Full-length gel images of western blot data in Fig. 2A and D.

Fig. 2H

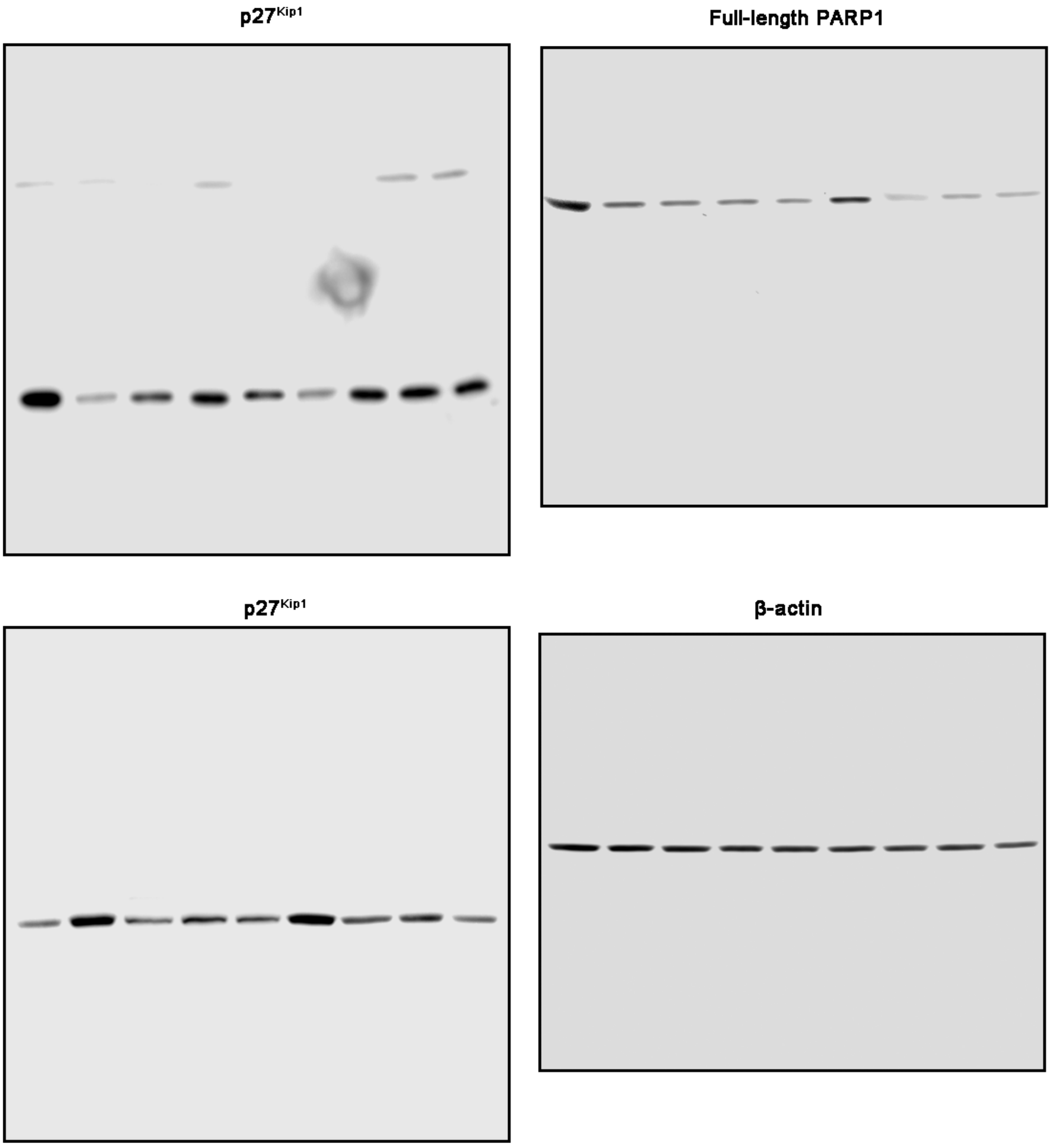

Supplementary Fig. 3. Full-length gel images of western blot data in Fig. 2H.
